# A taxonomy of states' approaches to plans of safe care

**DOI:** 10.1016/j.chiabu.2026.108169

**Published:** 2026-06-15

**Authors:** Margaret Lloyd Sieger, Sarah F. Loch, Barbara Andraka-Christou, Bradley D. Stein, Phoebe Levine, Lauren Caton, Stephen W. Patrick

**Affiliations:** a University of Kansas School of Medicine, Department of Population Health, Kansas City, KS, United States of America; b Emory University, Rollins School of Public Health, Department of Health Policy and Management, Atlanta, GA, United States of America; c University of Central Florida, School of Global Health Management & Informatics, Orlando, FL, United States of America; d RAND Pittsburgh, United States of America; e Emory University, Rollins School of Public Health, Emory Center for Child Health Policy, Atlanta, GA, United States of America; f Emory University, School of Medicine, Department of Pediatrics, Division of Neonatology, Atlanta, GA, United States of America

**Keywords:** Plans of safe care, Prenatal substance exposure, Child welfare policy, Policy scan

## Abstract

**Background:**

The 2016 Comprehensive Addiction and Recovery Act amended the Child Abuse Prevention and Treatment Act (CAPTA), expanding requirements for the development of state-specific *Plans of Safe Care* (POSC) for prenatal substance exposure. POSCs were intended to amplify existing efforts to include maternal referral to treatment and services in addition to infant safety planning. States were left to re-operationalize their own POSC procedures, including the extent to which the POSC process involved the child welfare system. We reviewed state statutes, regulations, and reports to create a taxonomy of state POSC approaches based on their relationship with child protective services (CPS) and healthcare systems.

**Methods:**

We evaluated state POSC-related statutes, regulations, and federally mandated child welfare state reports (Annual Progress & Services Reports, Child & Family Services Plans, & CAPTA plans) for all 50 states and Washington, D.C. from 2012 to 2024. Using artificial intelligence-assisted text extraction and subject matter expert textual interpretation, we reviewed 634 documents and more than 123,000 pages of text. Through iterative categorization, we developed a five-domain POSC taxonomy, organizing POSC policies by their proximity to and engagement with CPS and healthcare systems. We then identified the number of states with POSC policies in each domain and the timing of implementation.

**Results:**

Nearly all states had implemented their POSC by 2024. Our final taxonomy consisted of the following domains: (a) All CPS Referrals (26%), (b) Open Case Only (28%), (c) Alternative Response (26%), (d) Hospital-initiated (16%), and (e) Dual Pathways, that uses type of substance exposure to determine receipt of a Hospital-initiated POSC or traditional CPS response (14%). Eighty percent (80%) of states restrict POSC access to families already subject to CPS reporting or investigation.

**Conclusions:**

Despite federal intent to broaden public health supports, state POSC policies largely reinforce existing CPS practices rather than offering proactive, non-punitive interventions. The proposed taxonomy offers a framework for future comparative research, policy evaluation, and reform efforts.

## 1. Introduction

Perinatal substance use is a significant and growing public health concern. According to the National Survey of Drug Use and Health, in 2024, over 190,000 pregnancies in the U.S. (5.3%) were exposed to drugs (Center for Behavioral Health Statistics and Quality, 2025). Prenatal substance exposure (PSE) is a leading risk factor for child abuse and neglect (Austin et al., 2022; Goldstein & Font, 2025; Powell et al., 2025), sudden unexplained infant death (Lo et al., 2023; Piske et al., 2025), and maternal mortality (Han et al., 2024; Lisonkova et al., 2019). Up to 70% of children with PSE are referred to child protection systems by age 12 (Goldstein & Font, 2025; Powell et al., 2025), and 30 % of infants diagnosed with PSE at birth in California were placed into foster care before age 1 (Prindle et al., 2018). Infants with PSE who remain with their caregivers may be at increased risk of injury compared to peers placed into foster care (Austin et al., 2022; Palmer et al., 2025). These patterns reflect decades of federal and state policy efforts to address the needs of infants and families affected by substance use; efforts that have resulted in significant variation in how states respond to this population.

Federal child welfare law, the Child Abuse Prevention and Treatment Act (CAPTA), was amended in 2003 to improve the health and safety of infants with prenatal substance exposure (Lloyd et al., 2019). Since then, CAPTA has required states to have policies or procedures ensuring that healthcare providers involved in infant delivery “notify” Child Protective Services (CPS) of the birth and ensure the development of a “plan of safe care” (POSC) for infants “affected by” prenatal substance exposure. A POSC “generally entails an assessment of the family's situation and a plan for connecting families to appropriate services to stabilize the family and ensure the child's safety and well-being” (U.S. Government Accountability Office, 2018). For many years, little incentive or oversight existed for compliance with these elements of CAPTA and, as such, a minority of states developed POSC policies (Lloyd et al., 2019).

In 2016, in response to the opioid epidemic, the Comprehensive Addiction and Recovery Act (CARA) amended CAPTA, including adding a stated purpose of POSCs: to address the “health and substance use disorder treatment needs of the infant and affected family or caregiver.” This significant change shifted the focus beyond infant safety—the traditional role of CPS—to include the health and substance use treatment needs of both the infant and caregiver (Sieger et al., 2023).

The national child welfare system is guided by three concepts—safety, permanence, and well-being—meant to be held in tension with one another (Child Welfare Information Gateway, 2025; Holder, 2021). However, a traditional CPS response to prenatal substance exposure is often guided by a prioritization of safety, overriding the goals of permanence and well-being. This response typically includes four to five steps, based on the jurisdiction: (1) a referral to CPS from the community based on perceptions of risk, mandated reporting laws, or hospital policies, (2) a CPS decision to “screen-in” (or not) the referral based on statutory definitions of abuse or neglect or a risk assessment based on details included in the referral, (3) a CPS investigation into the veracity of the information included in the referral and other safety or risk factors, and (4) development of a safety plan that may include in-home child welfare services to address safety concerns or, if danger is perceived as great enough, (5) removal to foster care and court oversight (Child Welfare Information Gateway, 2025).

In prioritizing safety, this CPS-centric approach to prenatal substance exposure carries significant unintended consequences at odds with child health and well-being. Studies document that states relying on child welfare system involvement and foster placement for infants with PSE see reductions in women accessing prenatal care, substance use treatment during pregnancy, and medications for opioid use disorder, as well as increases in rates of neonatal abstinence syndrome (Atkins & Durrance, 2020; Austin et al., 2026; Bruzelius et al., 2024). Infants have higher rates of congenital syphilis in states with punitive PSE policies (Austin et al., 2025). One study documented greater developmental delays in infants with PSE placed into foster care compared to matched controls who remained at home (Novak et al., 2025). These punitive polices also fail to prevent substance use in pregnancy (Austin et al., 2026).

In contrast, a “public health approach” to child maltreatment prevention for this population would include early, universal screening, secondary prevention strategies for high-risk groups, and coordinated, multi-disciplinary care (Patrick et al., 2017). Many scholars and advocates believed CARA's changes to CAPTA offered an opportunity to expand this public health approach to infants with prenatal substance exposure (Lloyd Sieger et al., 2021). Aligning with CARA would mean state POSC policies should cover all infants affected by prenatal substance exposure and, at a minimum, ensure identification and referral to healthcare and treatment services for both caregiver and infant (Lloyd et al., 2019). Furthermore, federal CARA affords states leeway in deciding when a POSC is developed and by whom, including the opportunity to develop POSC before involving CPS, if CPS is involved at all (Child Welfare Information Gateway, 2020b).

Unfortunately, a 2018 policy scan found few states' POSC approaches aligned with the parent-child dyad language in CARA (Lloyd et al., 2019). In most cases, states responded to CARA's changes by implementing new CPS reporting mandates or retitling their traditional safety planning process a “plan of safe care” (Lloyd et al., 2019). Simply retitling traditional child protection activities has been viewed as a missed opportunity to institutionalize an upstream, public health approach to supporting families caring for an infant with prenatal substance exposure (Lloyd et al., 2019; Lloyd Sieger et al., 2021; Patrick et al., 2017; Patrick et al., 2019).

However, a few states did use CARA's passage as an opportunity to innovate in a public health direction. New Mexico, for example, codified in statute that prenatal substance exposure alone does not require a CPS report; instead, healthcare providers offer voluntary POSCs with care coordination through health insurers—not the child welfare system—connecting families to medical, substance use, and social services (NM Health; New Mexico Department of Health et al., 2022; Sharp et al., 2023). The implementation experience of these innovating states, while instructive, has not been systematically documented or compared across the broader national landscape.

The current state of POSC policies nationally is poorly understood. A recent policy surveillance study found that, while only 18 states had statutes or regulations about POSC, all but one state has implemented POSCs in some form (Lloyd Sieger et al., 2025). That study highlighted significant challenges in accessing states' POSC policies, in part because states use a variety of terms for their POSC policies and, despite a federal mandate, do not make current and historic state reports readily available for public inspection (Lloyd Sieger et al., 2025).

A lack of systematic, national accounting of POSC approaches has hindered evidence-based policymaking and comparative research. The literature also lacks a taxonomy of common POSC approaches; a precondition for empirically assessing the effects of alternative policy types. Such a taxonomy is urgently needed as CAPTA approaches federal reauthorization (Child Welfare Information Gateway, 2019), and policymakers need information to guide POSC changes. Therefore, the purposes of this paper are to: 1) review each state's stated approach to POSC, 2) develop a taxonomy of states' stated POSC approaches, and 3) assign each state to a category within this taxonomy.

## 2. Methods

### 2.1. Data collection

To build a dataset of brief descriptions of POSC policies from 50 US states and Washington D.C. (collectively “states”), we used a combination of findings from our recently published legal review of state statutes and regulations (Lloyd Sieger et al., 2025), as well as a content analysis of publicly available state reports submitted to Congress annually. Previous studies on POSC have leveraged these reports to document states' POSC policies (Lloyd et al., 2019). We describe our data collection process in more detail below.

### 2.2. State statutes and regulations

The current analysis builds on our policy surveillance of POSC statutes and regulations (Lloyd Sieger et al., 2025). As described in our prior paper (Lloyd Sieger et al., 2025), we iteratively developed and applied a search string of POSC-related terms in Nexis Uni legal software, screening state statutes and regulations in effect as of November 2024. Two members of the research team independently evaluated each law for inclusion based on defined inclusion/exclusion criteria. Policies were included if they described the state's approach to “plans of safe care,” defined as a systematized, statewide process for connecting infants with prenatal substance exposure and their caregivers to support services (either within or outside CPS) with a goal of ensuring health, safety, or well-being. Policies were excluded if they only applied to licensed substance use disorder (SUD) facilities, only applied to children older than infants, only applied to neonatal abstinence centers or residential infant care centers, only restated federal requirements, only discussed requirements related to the federal Individuals with Disabilities Education Act rather than CAPTA, only described requirements for policymakers or task forces to make future plans (e.g., to assess the feasibility of a hypothetical future program), or used relevant terminology in the wrong context (e.g., discussed plans to keep children safe from violence in residential facilities). Eighteen states (35% of 51) had statutes or regulations that met the inclusion criteria. For the current study, we collected historical versions of the laws described above, resulting in a dataset of laws from 2012 to 2024 matching our original inclusion/exclusion criteria.

### 2.3. State reports

Based on the findings from our first study (Lloyd Sieger et al., 2025), we concluded that most states did not have POSC statutes and regulations, and those that did often provided few details of states' POSC policies. Therefore, we also compiled all 51 states' Annual Progress & Services Reports (APSR), Child & Family Services Plans (CFSPs), and CAPTA State Plans (hereafter collectively “State Reports”), which are mandated state reports to Congress of federally funded child welfare activities, including POSCs, from 2012 to 2024. Between January and June 2024, we searched for State Reports online and via email, locating 630 documents, totaling 123,142 pages of text. A complete list of these documents is included in the supplement.

### 2.4. Analysis

#### 2.4.1. AI extraction

Given the large number of State Reports that could contain pertinent information, we leveraged several artificial intelligence tools to reduce the volume of text requiring human review. We first used the Instructor Python library (Liu & Contributors, 2024) to extract structured output from the unstructured text of each page using the following process. For each page containing at least one keyword, we showed the full text to GPT-4.0-mini and asked it to return a JSON (Javascript Object Notation; an open data interchange format that is both human and machine-readable) that documented each keyword mentioned on the page. We defined the JSON structure to have fields for keyword, the exact sentence containing the keyword, and the exact text of any surrounding relevant sentences. For each State Report page, we then showed all extractions to GPT-4.0-mini in a single prompt and asked it to summarize the document's key points relating to plans of safe care, organizing the summaries and extractions from each document into state-level files sorted by year for review. Prompts and model parameters are included in our supplement.

#### 2.4.2. Identification of target passages

Next, two authors (M.L.S. and S.F.L.) identified target passages in statutes, regulations, and State Reports, focusing on text describing the state's current POSC processes. These target passages characterized as many dimensions of the state POSC process as possible, such as which infants received POSC, who completed the POSC, and the services focus of the POSC.

We took several steps to identify target passages. First, relevant content from statutes and regulations was manually extracted by two POSC experts, as previously described (Lloyd Sieger et al., 2025). Next, AI-extracted text from the State Reports were reviewed by the same two POSC experts (M.L.S. and S.F.L.) to locate the first mention of the states' POSC approach, and any subsequent mentions, to identify possible evolution of the policy. The statewide POSC process was most often described in one to three paragraphs in the State Report text, paragraphs that were often repeated, in whole or in part, in subsequent years' documents. We excluded states' discussions of planning, pilot projects, partially implemented statewide programs, or future goals because the durability of these plans and pilots is unknown and would not influence the child welfare responses to infants with PSE statewide. If the extracts did not provide enough detail, or if we needed additional context to understand an extract's meaning, we reviewed the original document from which the extract came. In a few cases, if the State Report used an unfamiliar term or acronym, the meaning of which was necessary to understand the states' POSC approach, we searched for the term online. For a few documents, we read the embedded links or references to enhance our understanding of the text.

If the State Report provided more detailed information on the POSC process than did the statute or regulation (among the 18 states with statutes or regulations), we selected the State Report text as the target passage. In sum, 9 states' (17.6%) had target passage text from statutory language, and 41 states (80.4%) had target text from State Reports. One state had no policy at the time of our analysis (November 2024). The specific target passages and their source citations are included in the supplement.

In addition to characterizing states' POSC process, we assigned an effective date. For statutes and regulations, we relied on the effective date; if no such date was available, we relied on the enacted date. State Reports frequently mentioned a specific date that the POSC approach was implemented. If the target passage or surrounding text did not reference a particular start date, we chose the year the document containing the target passage was published.

#### 2.4.3. Generating & applying POSC taxonomy

Following extensive iterative review of the extracted text and coding of statutes and regulations completed by two subject matter experts (M.L.S. and S.F.L.) and a legal expert (B.A.C.) (see Lloyd Sieger et al., 2025), we developed a taxonomy to deductively categorize each state's POSC policy. Consistent with qualitative framework analysis applied to policy documents (Gale et al., 2013; Ritchie & Spencer, 2002), taxonomy development was an inductive, expert-driven process in which conceptual categories emerged through systematic review and iterative refinement rather than algorithmic procedures. An initial codebook consisted of three domains — traditional CPS, hybrid approach, and hospital-initiated — derived from prior literature (Lloyd et al., 2019; Lloyd Sieger et al., 2021; Lloyd Sieger et al., 2022; Lloyd Sieger & Rebbe, 2020) and subject-matter expertise. As independent review proceeded, it became clear that the initial structure did not adequately capture meaningful variation within CPS-embedded approaches: specifically, states differed in *when*, within the continuum of child welfare services, families received a POSC: at referral regardless of case opening, only upon opening a CPS case, or through a distinct alternative response pathway. A fifth domain emerged from two states whose policies were not accommodated by any existing category. The final taxonomy was organized with respect to the proximity of the POSC process into and through child welfare system activities, and domain names were iteratively revised based on input from co-authors and community advisors throughout.

Following taxonomy development, two subject matter experts (M.L.S. and S.F.L.) then independently applied the five domains deductively to each state's target passage, treating the taxonomy as a fixed framework. This second stage was distinct from and sequential to the inductive development process: separating domain construction from domain application was intentional, to avoid the circular reasoning that would result from simultaneous category development and assignment. AI was not used during this process. Following initial review, the two experts agreed on the categorization of 72% (*n* = 36) of states. The experts then discussed all states where there was disagreement (*n* = 14) and ultimately reached consensus for 100% of states. Using a consensus building approach is favorable when coding complex, ambiguous, or nuanced legal texts as was the case in this study (Burris, 2014). The domain names within the taxonomy were iteratively revised based on input from co-authors and community advisors. We include a list of each state, the target passage, the assigned category, and the effective date in our supplement.

## 3. Results

### 3.1. POSC taxonomy

**Fig. 1.**
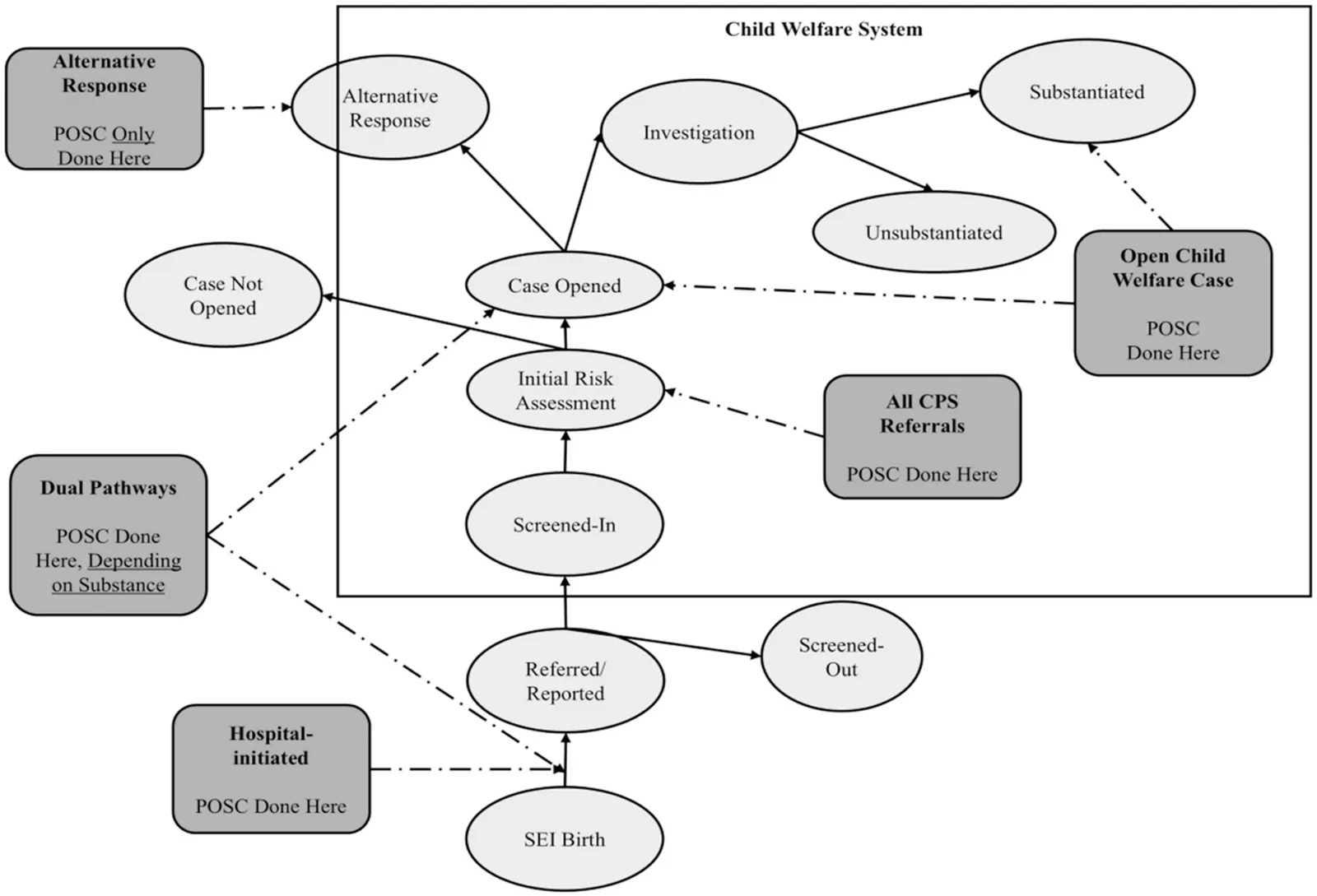
Taxonomy of States' POSC Policies Relative to Substance-Exposed Infant Birth and the Child Welfare System Fig. 1 Notes. POSC = plan of safe care. SEI = substance-exposed infant. Each light gray oval represents an event for an individual family of an infant with prenatal substance exposure. Solid arrows demonstrate how each event is connected in a typical process. Events within the large square occur within the context of the child welfare system. The cornerless rectangles represent each of the five types of state POSC approaches according to our taxonomy, which are differentiated based on when in a series of events the POSC is completed. The timing of POSC completion for each theme is illustrated with the dashed lines. Bolded text indicates the title of the theme.

Fig. 1 shows the five taxonomy domains and surrounding context.

#### 3.1.1. Domain: hospital-initiated

The domain most proximal in time to the infant's birth is the Hospital-initiated POSC. States using this approach require a POSC to be developed in pregnancy or shortly after birth, outside the context of the child welfare system, before a CPS referral decision is made. Because the POSC is developed in pregnancy or around the time of delivery, healthcare providers (including hospital-based social workers or nurses) or other community-based professionals develop the POSC. States that leverage this approach typically do not have a CPS reporting mandate for prenatal substance exposure, but may have a separate monitoring system created to track the number of infants and families receiving POSCs for federal reporting. With this approach, if a report is made due to safety concerns, the CPS response and any safety planning are distinct from the POSC. Because the POSC is developed outside the context of the CPS system before any potential referral is made, the plan functions as a secondary public health intervention designed to provide enhanced, targeted supports to a high-risk group.

#### 3.1.2. Domain: dual pathways

States with a Dual Pathways response use a Hospital-initiated POSC for some specific substance exposures (e.g., medications for opioid use disorders) but mandate a referral and CPS response for other specific exposures (e.g., any illegal or illicitly obtained controlled substance). POSCs in this context, although supportive and community-based, are only available to certain parent-infant dyads. Furthermore, these states use the type of substance exposure as a screening tool for determining POSC eligibility.

#### 3.1.3. Domain: all CPS referrals

Moving downstream, some states have employed POSC for all screened-in referrals involving infants with prenatal substance exposure, the “All CPS Referrals” response. In these states, accessing a POSC requires a screened-in report, meaning CPS has decided the parent or child meets prenatal substance exposure criteria. States using this approach often mandate a CPS report for prenatal substance exposure and then require the POSC to be developed during the risk assessment (or investigation, depending on the state) process, regardless of whether the family will have a child welfare case opened or not. For families that have a case opened, the POSC is often incorporated into their subsequent safety plan. For families that do not have a case opened, the POSC then belongs to the family for use in the community. Although similar to a Hospital-initiated POSC, because of its universality, the POSC exists within the context of the CPS system and is therefore distinct. For families with past CPS involvement, this new referral significantly increases the likelihood that a new case would be opened. For families without prior CPS involvement, this plan may unnecessarily expose them to the CPS system if a case is ultimately not opened.

#### 3.1.4. Domain: alternative response

States employing this approach have created an Alternative Response (also known as a differential response, dual track, or family assessment) pathway or leveraged an existing differential response pathway for cases that meet criteria to receive a POSC. These states lean into the supportive, voluntary aspects of a Hospital-initiated approach, but do so largely within the context of the CPS system. “Differential response” was originally developed as a second “track” within child welfare to address families referred to CPS for help meeting basic needs or other lower-risk concerns for which a child maltreatment investigation was not appropriate or warranted (Child Welfare Information Gateway, 2020a). Differential response services are always voluntary, delivered “in-home” (meaning, without parent-child separation), and administered without court involvement (Child Welfare Information Gateway, 2020a). For states using the Alternative Response approach, most use in-home services provided or overseen by the state's child welfare system or contracting agency. However, a few states connect families to services provided or overseen by entities outside the CPS system, such as the state's health department.

#### 3.1.5. Domain: open child welfare case

Finally, the approach that aligns with the deepest child welfare system penetration is the Open Child Welfare Case response. These states implement POSC only after a family is deemed in need of child welfare intervention. In every practical sense, a POSC in these states is a child welfare safety plan or case plan. Parents' failure to comply with the POSC in these states would likely have significant consequences, including punitive measures, such as child removal or termination of parental rights.

### 3.2. Distribution of states by domain

**Fig. 2.**
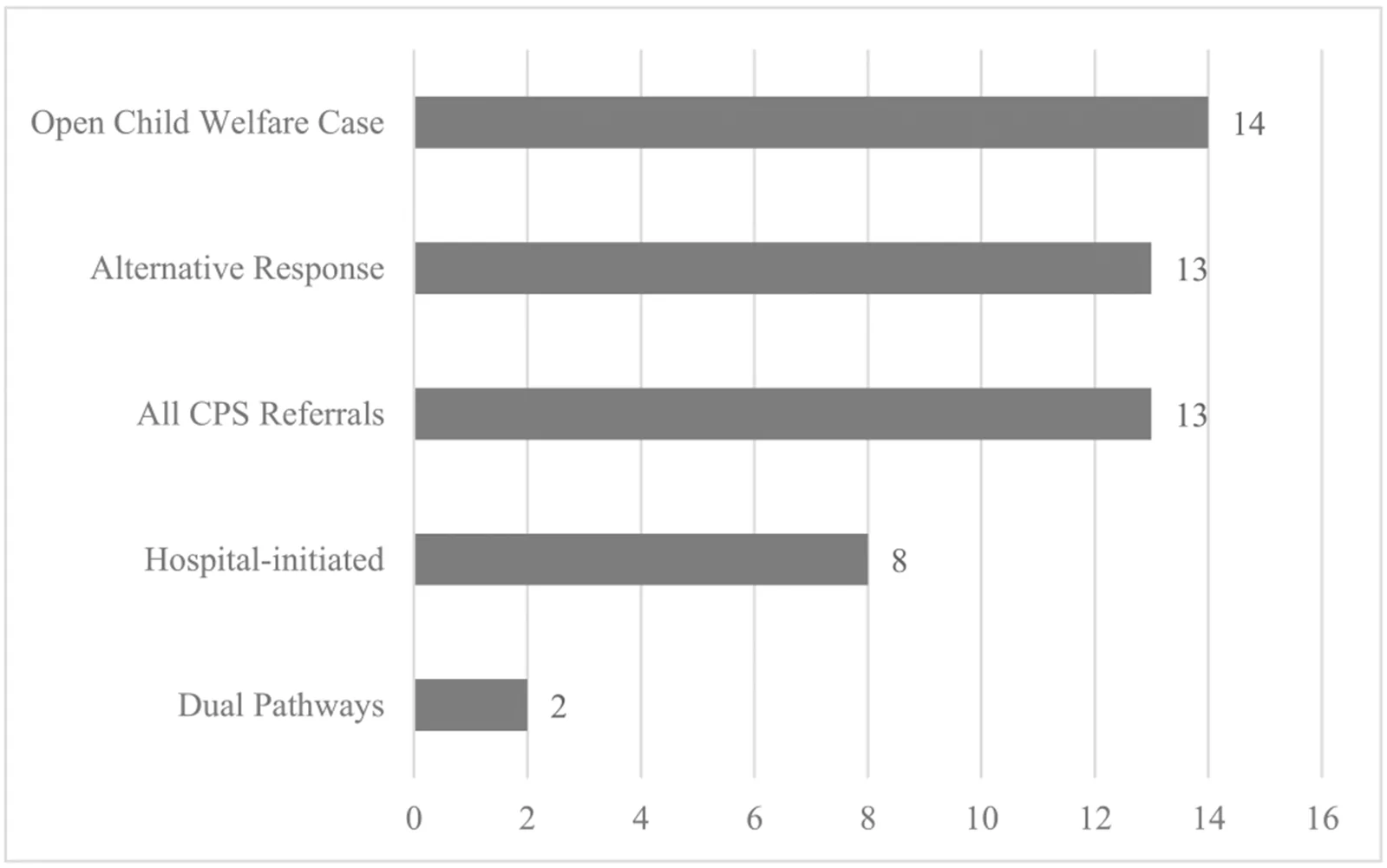
Frequency of 50 States' POSC Policy Domains as of 2024 Fig. 2 Notes. POSC = plan of safe care. Traditional/Open = Traditional Approach, Open Cases Only. Traditional/All = Traditional Approach, All Referrals. Each bar illustrates the number of states (including D.C.) whose POSC policies align with each of the five POSC domains in our taxonomy of POSC approaches as of 2024. These domains differ based on when the POSC is completed. A complete description of each theme is included in our manuscript text. Illinois did not have a statewide POSC approach in place by 2024 and thus is excluded from Fig. 2 frequencies.

Every state except Illinois implemented some type of POSC approach by 2024. Illinois started a task force on “family recovery plans implementation” that first convened in April 2025 (Illinois Department of Human Services, 2025). Fig. 2 illustrates the distribution of states whose POSC approaches align with each domain. The most common, Open Child Welfare Case, was implemented in 14 states (28%), with All CPS Referrals and Alternative Responses, each implemented in 13 states (26%). Eight states (16%) implemented Hospital-initiated approach, and two states (4%) implemented a Dual Pathways approach.

**Fig. 3.**
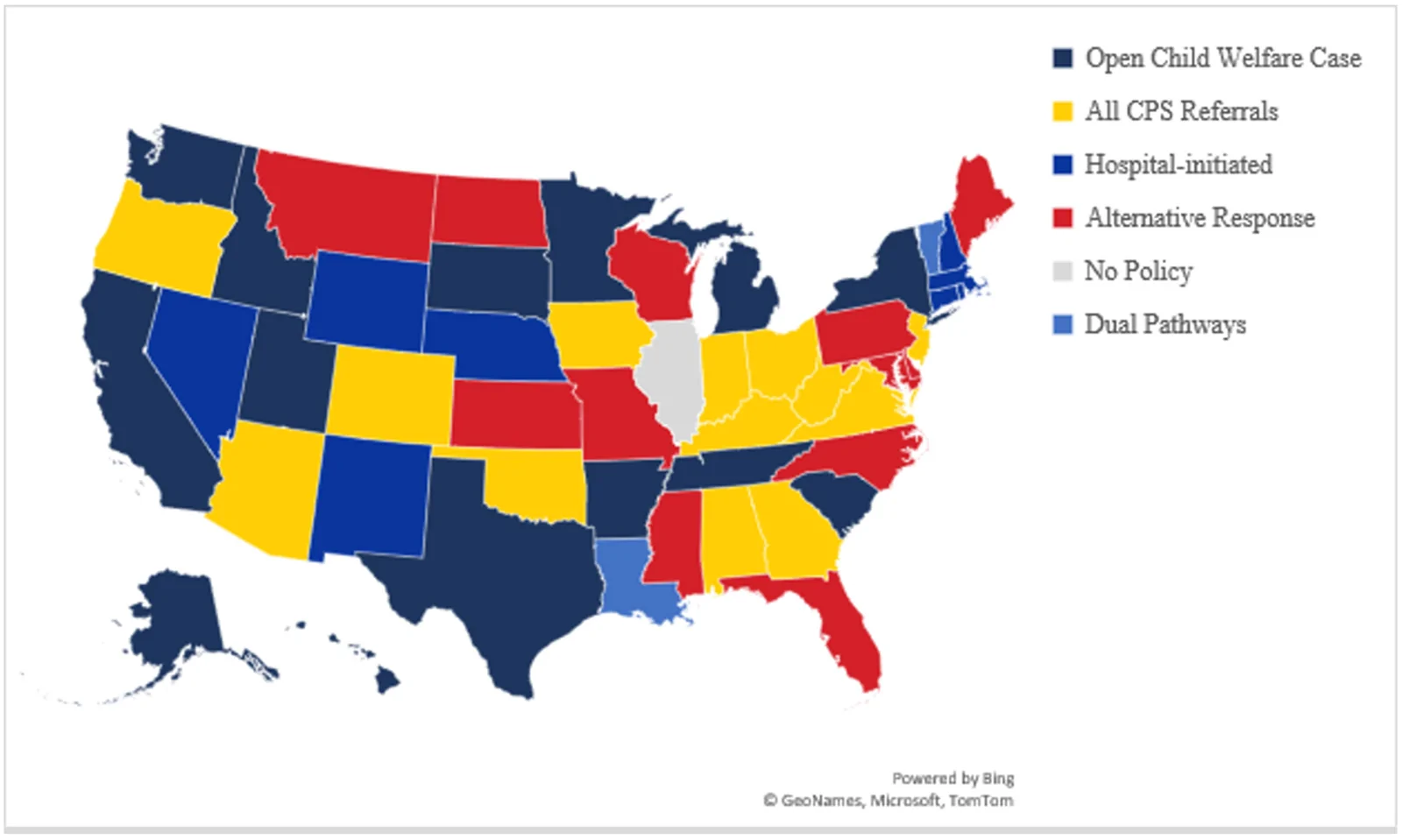
Map of 51 States' POSC Policy Domains as of 2024 Fig. 3 Notes. POSC = plan of safe care. Traditional/Open = Traditional Approach, Open Cases Only. Traditional/All = Traditional Approach, All Referrals. Each color illustrates a POSC policy theme according to our taxonomy. These domains differ based on when the POSC is completed. A complete description of each theme is included in our manuscript text. Illinois did not have a statewide POSC approach in place by 2024 and thus is coded as “No Policy” above.

Fig. 3 illustrates each states' approach, as well as some regional clustering, particularly for states using the All CPS Referrals and the Hospital-initiated approaches.

**Fig. 4.**
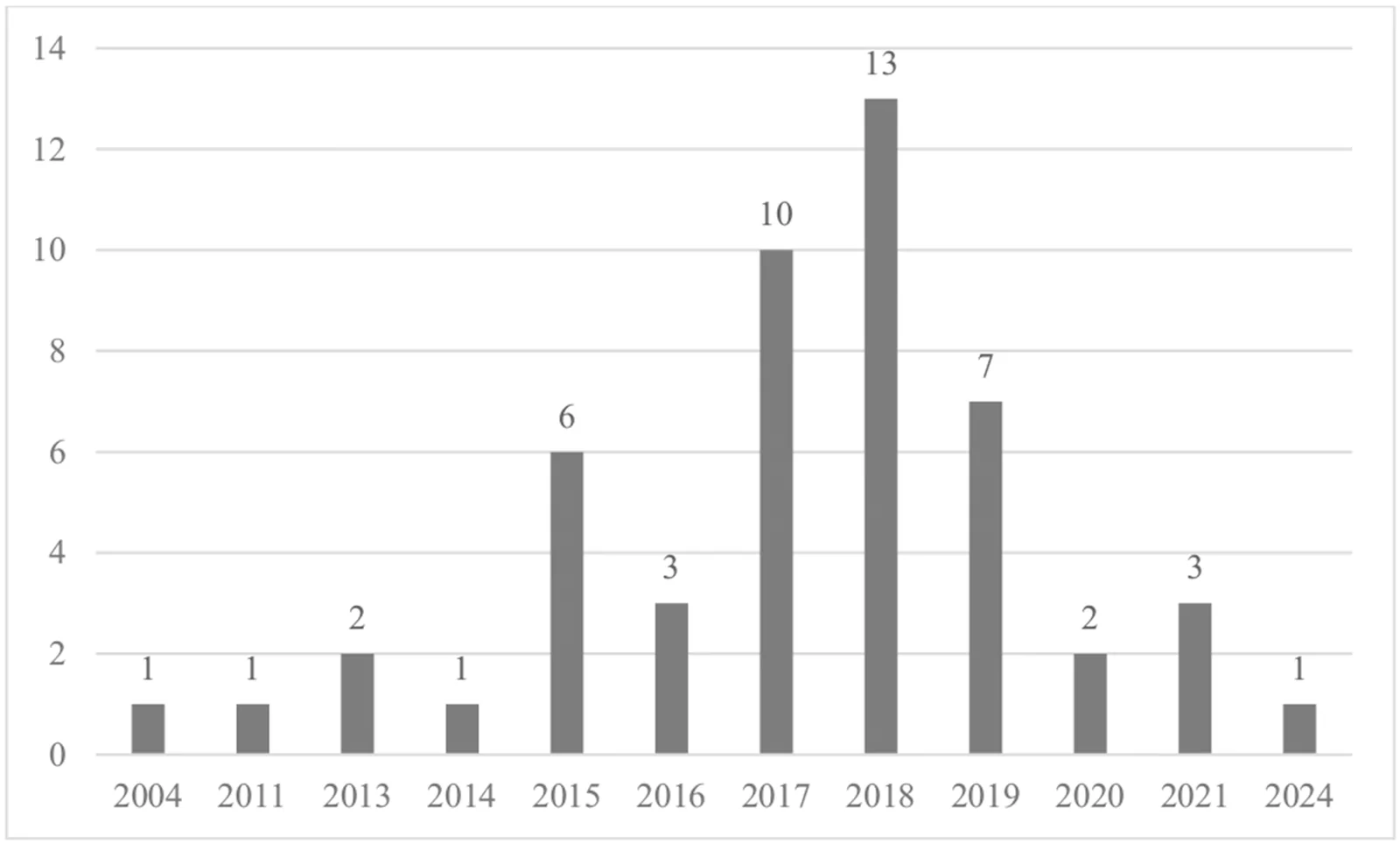
Effective Date of 50 States' Statewide POSC Policies as of 2024 Fig. 4 Notes. POSC = plan of safe care. Each bar illustrates the number of states (including D.C.) whose POSC approach was installed in each depicted year. These dates come from a variety of sources varying in specificity ranging from statutes/regulations with a specific enact date, to publication year of the report to congress describing the state's POSC approach. Illinois did not have a statewide POSC approach in place by November 2024 and thus is excluded from Fig. 4 frequencies.

Lastly, Fig. 4 summarizes the distribution of effective dates as of 2012, the earliest date we searched for policies, illustrating that the most common effective dates for POSC policies were between 2017 and 2019, with 60 % of states' policies effective during these three years.

## 4. Discussion

For more than twenty years, federal policy has required states to use POSC in responding to infants with prenatal substance exposure; however, most states did not have a POSC approach in effect until after CARA's 2016 passage. Over time, states have adopted a variety of POSC approaches, but no previous research has articulated a taxonomy for categorizing states' POSC policies, a precondition for empirically assessing the effects of different types of policies. This has contributed to a paucity of empirical research examining whether and how POSC policies can improve outcomes for infants, parents, and families.

We previously documented the many barriers involved in inspecting states' POSC approaches (Lloyd Sieger et al., 2025). Employing a variety of tools to identify a description of each state's POSC approach, generate a taxonomy, and apply the taxonomy to states' policies, we extend that research, identifying state policies by reviewing 123,000 pages of text to identify only a few paragraphs total per state of relevant information. This is the proverbial “needle in a haystack.” To find these needles, we leveraged a commercial large language model, a tool that proved immensely helpful at reducing the size of the haystack but still required extensive expert review and critical analysis to interpret legal jargon in context and to categorize the legal data. Future research on POSCs may increase in rigor if primary data from state child welfare agencies is collected systematically to capture some of the granular details that were difficult to glean from the State Reports, or if states were to make these policies publicly available outside of state reports.

We extended past research by creating a POSC taxonomy. Existing literature on POSC has pointed to two approaches: a (rare) public health approach delivered outside the context of CPS, and a widespread traditional CPS-based approach (Lloyd et al., 2019; Lloyd Sieger et al., 2025; Sieger et al., 2022). We distilled five different types of approaches, differentiated by their relationship with the trajectory into and through the child welfare system, identifying nuance between the two extremes. Although states' approaches differ in countless other ways, such as which state partners are assigned/identified, training frequency, funding streams, modes of documentation, as well as a variety of ongoing pilot projects, our taxonomy reflects decision-points in CPS trajectories likely to have the largest impact on focal outcomes, including increasing healthcare access, preventing child maltreatment, and keeping parents and children together whenever possible.

Our taxonomy illustrates that most states have restricted POSC access to families who get reported to CPS and, in many of these states, only families whose maltreatment reports result in opening a child welfare case, frequently after substantiating a maltreatment allegation. Rates of substantiation vary considerably across states, often reflecting differences in legal and organizational contexts rather than the “facts” of maltreatment (Font & Maguire-Jack, 2021). Although many of these cases will involve court-ordered service plans and out-of-home placement, not all will (Font & Maguire-Jack, 2021). A new study from Pennsylvania examining linked CPS and Medicaid data for families with a CPS case between 2015 and 2018 found that the extent of CPS system penetration was significantly associated with whether the child's mother accessed SUD treatment (Goldstein & Font, 2025). Specifically, mothers whose children were placed into foster care were significantly more likely to enter treatment than mothers receiving in-home services. The Pennsylvania study did not focus on cases involving infants with prenatal substance exposure or families with a POSC; a subgroup analysis pointed to more tempered effects for cases involving children under one year old (Goldstein & Font, 2025). Notably, Pennsylvania uses an Alternative Response approach, suggesting that the differential response pathway for POSC may increase connection to treatment for mothers of infants compared to families with older children receiving traditional in-home services. Nonetheless, the Pennsylvania study suggests that traditional child welfare intervention may motivate (or force) parents to enter substance use treatment (Goldstein & Font, 2025). No research has specifically examined the effects of POSC policies in a state that uses traditional child welfare-based approaches (i.e., All CPS Referrals or Open Case Only).

We also found that eight states used a Hospital-initiated approach to POSC, an approach less traditional than approaches embedded in the child welfare system. Research on Connecticut and New Mexico, two states using such an approach, suggests that most but not all eligible families receive POSC (Sharp et al., 2023; Sieger et al., 2023). New Mexico, in particular, has faced considerable recent scrutiny (Riley, 2025), as it assigns the responsibility of POSC development to case managers embedded in health insurance systems. Although the New Mexico policy also integrates “CARA Navigators” in the CPS system (i.e., CPS case managers assigned to work with prenatal substance exposure cases), media and advocates have characterized the voluntary POSC as causing several high-profile infant deaths (New Mexico Legislative Finance Committee, 2023; Riley, 2025; Sharp et al., 2023). However, CPS systems that respond to such tragic events by increasing the foster care placement rates do not impact the rate of child fatalities (Heath, 2024), as such death are incredibly rare, difficult to predict, and often occur after the family is known to CPS. On the other hand, Connecticut, which also uses a Hospital-initiated approach and has also observed reduced rates of reports and foster care placement among infants with prenatal substance exposure (Sieger et al., 2025), has ostensibly increased the precision of the pre-referral process (i.e., the hospital workers' decision making), thus ensuring that high maltreatment risk cases are still connected to CPS (Sieger et al., 2022), while lower risk families are referred for supports elsewhere. Future research using causal inference approaches will aid in understanding the extent to which these POSC schemes impact the health and safety of infants.

The Dual Pathways approach, only in Vermont and Louisiana, was unique, warranting its own categorization. In these states, infants exposed only to certain substances receive a Hospital-initiated POSC with no CPS contact, while all other types of prenatal substance exposure result in a CPS referral for safety planning with the child welfare department. Although not explicitly stated, the forms of prenatal substance exposure are eligible for a POSC are considered low risk from a CPS standpoint. In Vermont, POSC-eligible infants are those exposed to cannabis, which appears not to increase risk for subsequent maltreatment, (Ryan et al., 2025) or appropriately used controlled medications (e.g., pain medications, psychotropics). Similarly, in Louisiana, infants are eligible for POSC only if the infant is born affected by controlled substances used in a lawfully prescribed manner. The Dual Pathways approach does three novel things: 1) it leverages the type of substance exposure as a proxy for safety, 2) it protects parents appropriately using medications from unnecessary CPS intrusion, and 3) it offers additional support to these low-risk families outside the context of the child welfare system. These elements address some criticisms of CAPTA, including concerns related to CPS involvement when parents are receiving medications to treat opioid use disorder (MOUD) (Lloyd et al., 2019). Leading professional organizations have made calls for exceptions to CPS reporting for certain medications (American Psychological Association, 2020; Guyer et al., 2024). To date, no studies exist on the effects of Vermont's or Louisiana's approach.

States implementing an Alternative Response approach have implemented a distinct, voluntary, in-home services pathway for infants with prenatal substance exposure. Historically, younger children have been *less* likely to receive these services (Damman et al., 2020). This likely reflects many factors including their increased physical vulnerability. Furthermore, parents receiving voluntary services may access less substance use treatment compared to parents whose children were placed into foster care (Goldstein & Font, 2025), and infants with substance-related allegations who remain at home may be at higher risk of subsequent injury compared to their peers in out-of-home placement (Palmer et al., 2025). Research from Delaware, a state using an Alternative Response approach, suggest that concerns for parental disengagement and lack of infant safety with in-home POSC may be unfounded (Deutsch et al., 2022). Ninety-four percent of the Delaware study sample (i.e., infants with prenatal substance exposure reported to CPS) received a POSC, suggesting high levels of implementation and engagement. The majority (88%) remained at home (Deutsch et al., 2022), suggesting high levels of safety and, if safety became a concern, appropriate increase in intensity of response (i.e., out-of-home placement). Seven infants in the study sample (0.51%) sustained serious injuries and four infants died (0.30%). This fatality rate is equivalent to 2.7 per 1000 births. For comparison, the national infant mortality rate in 2022 was 5.1 per 1000 births. (Ely & Driscoll, 2024).

### 4.1. Limitations

This study has several limitations. We relied on publicly available documents that could, but were not required to, describe states' POSC processes, and did not have complete records from any state, potentially resulting in important omissions regarding states' policies or practices. Relying on a secondary accounting of each state's approach likely impacted the accuracy of our measurement. We engaged in an iterative process of information gathering and analysis that was difficult to systematically describe, which may impact replicability. We also focused just on POSCs, not including in our analysis states' laws on mandated reporting, definitions of maltreatment, screening, investigation, and substantiation criteria, and other legal and organizational characteristics that likely influence families' entry into and through the child welfare system. We do not know how this information would influence our findings. Finally, we excluded pilot projects or new programs not implemented statewide at the time of our analysis (or based on the most recent State Report we received), and such pilot projects or new programs may reflect more innovative approaches to POSC.

### 4.2. Conclusion

We offer a taxonomy organizing states' approaches to Plans of Safe Care. Our results suggest most states have integrated POSC into traditional CPS practice. Doing so may fall short of goals to implement upstream, interdisciplinary, and collaborative public health interventions for families with substance use in pregnancy; it likely reflects that POSC are embedded in federal child welfare legislation, tied to very modest federal child welfare funding, and implemented populations historically requiring high rates of CPS intervention. Furthermore, limited evidence exists that *any* POSC approach “works” at the individual level to increase parent or infant receipt of healthcare or substance use treatment services or reduce child maltreatment. Without financial or outcomes-based incentives to change, many CPS systems—historically challenged by high needs and limited resources—appear to maintain the status quo. That said, as of 2024, twelve states are implementing approaches outside child welfare (i.e., Hospital-initiated or Dual Pathways) for either all or some infants with prenatal substance exposure. Studies examining the process and individual and system-level effects of these approaches are urgently needed to inform pending federal legislation revisions and better understand systematic differences in and effects of POSC policies.

## Supplementary Material

Appendix A

Appendix B Supplementary data

## Data Availability

We have shared the data in our supplement.

## Article summary

We analyzed states' statutes, regulations, and reports to Congress to develop a taxonomy of approaches to Plans of Safe Care (POSC) for infants with prenatal substance exposure, organized around extent of involvement of child protective services (CPS). Three of the five domains, constituting 80% of states, use POSC approaches that “live” inside CPS. Ten states, in two policy categories, implement POSC outside CPS.

## What's Known on this Subject

Federal law requires state policies or procedures on POSC for infants with prenatal substance exposure. Only 18 states have statutes or regulations on POSC; the majority enact this approach in administrative policy.

## What this Study Adds

Our taxonomy provides a useful approach for categorizing state POSC policies with respect to their relationship to CPS or healthcare services. Most states' POSC policies involve CPS, potentially resulting in punitive approaches contrary to the intent of federal law.

## Appendix A

**Appendix Table 1:** Keywords Used in APSR Search.

safe care plan	exposed	sei
safe plan of care	exposure	sen
posc	expose	sei-
plan of care	withdrawal	sen-
plans of care	neonat	sei.
plan of safe care	perinatal substance	sen.
plans of safe care	prenatal substance	substance exposed newborn
plan for safe care	perinatal drug	substance-exposed newborn
plans for safe care	prenatal drug	substance exposed infant
plan for the care	substance affect	substance-exposed infant
plans for the care	family wellness support plan	infant care plan
plan for the safe care	family wellness and support plan	family care plan
plans for the safe care	cara plan of care	

Note: Searches were not case-sensitive.

**Appendix Table 2:** Page-level LLM Extraction Details.

Model	gpt-4.0-mini
Temperature	1.0 (default) Act as an expert in plans of safe care policy\n\n A Plan of Safe Care is designed to ensure the safety and well-being of an infant with prenatal substance exposure by addressing the health and substance use disorder treatment needs of both the infant and the affected family or caregiver. \n\n A Plan of Safe Care has been shown to reduce child safety risks related to substance use disorders within a family and therefore is an effective strategy for keeping families safely together and preventing the need for foster care. \n\n For infants with prenatal substance exposure, Plans of Safe Care bring together affected families and various service providers—including maternal and infant healthcare, substance use disorder treatment, mental health treatment, early childhood, child welfare. \n\n This collaborative approach ensures a comprehensive response that prevents crisis-based reactions, ensures infant safety, promotes healthy child development, and strengthens family protective factors.\n\n Since 2003, there have been three significant changes in requirements about implementing Plans of Safe Care. There is great variation among states, both in terms of the implementation of their policy and procedures and the rate of infants placed in out-of-home care due to substance use disorder-related issues.\n\n The Keeping Children and Families Safe Act of 2003 created new conditions, including the Plan of Safe Care, for states to receive grant allocations under the Child Abuse and Prevention Treatment Act (CAPTA). The legislation required states receiving a CAPTA grant to implement policies and protocols for the following:\n\n
System Message	- Appropriate referrals to child protection services and other relevant systems to address the needs of infants born with and identified as affected by illegal substance abuse or withdrawal symptoms resulting from prenatal drug exposure.\n\n - A requirement that health care providers involved in the delivery or care of such infants notify the child protective services agency of the occurrence of such condition except that such notification shall not be construed to establish a definition under federal law of what constitutes child abuse or require prosecution for any illegal action.\n\n - The development of a Plan of Safe Care for the infant born with and identified as being affected by illegal substance abuse or withdrawal symptoms. \n\n You are an expert at searching text for the **EXACT** strings of keywords in {list of keywords from Appendix Table 1piped in here} and returning the **EXACT** sentence and text that the keyword was mentioned in\n\n If a sentence has multiple keywords in it, record the sentence once; do **NOT** record it for every keyword
Prompt	Please review the following text: {text from APSR page piped in here}\n\n Then return any sentences containing the keywords provided in your system message as an ‘ExtractMentions’ object Pydantic BaseModel object with two attributes:
ExtractMentions Object	sentences: list of KeywordSentence objects; described to the model as “list of unique KeywordSentence objects which represent sentences containing keywords that you find in this text” summary: string; described to the model as “summary of the texts key points, state actions, or themes related to plans of safe care” Pydantic BaseModel object with three attributes:
KeywordSentence Object	keyword: keyword chosen from keyword list in Appendix Table 1; described to model as keyword that you found in the sentence; pick the first keyword if there are multiple” sentence: string; described to model as “exact, full sentence containing keyword” context: string; described to model as “exact text, including any relevant surrounding sentences that provide context, containing keyword”

## Appendix B. Supplementary data

Supplementary data to this article can be found online at https://doi.org/10.1016/j.chiabu.2026.108169.
