# Supplementary material for: A taxonomy of states' approaches to plans of safe care
Source: Child Abuse Negl. Author manuscript; Available in PMC 2026 Sep 8. (PMC13551497; doi:10.1016/j.chiabu.2026.108169)
Supplement: Supplementary file 1 [file NIHMS2205323-supplement-Appendix_A.docx]

Appendix A.

Appendix Table 1. Keywords Used in APSR Search.

| **safe care plan** | exposed | sei |
| --- | --- | --- |
| **safe plan of care** | exposure | sen |
| **posc** | expose | sei- |
| **plan of care** | withdrawal | sen- |
| **plans of care** | neonat | sei. |
| **plan of safe care** | perinatal substance | sen. |
| **plans of safe care** | prenatal substance | substance exposed newborn |
| **plan for safe care** | perinatal drug | substance-exposed newborn |
| **plans for safe care** | prenatal drug | substance exposed infant |
| **plan for the care** | substance affect | substance-exposed infant |
| **plans for the care** | family wellness support plan | infant care plan |
| **plan for the safe care** | family wellness and support plan | family care plan |
| **plans for the safe care** | cara plan of care |  |

Note: Searches were not case-sensitive.

Appendix Table 2. Page-level LLM Extraction Details.

| **Model** | gpt-4.0-mini |
| --- | --- |
| **Temperature** | 1.0 (default) |
| **System Message** | Act as an expert in plans of safe care policy\n\n A Plan of Safe Care is designed to ensure the safety and well-being of an infant with prenatal substance exposure by addressing the health and substance use disorder treatment needs of both the infant and the affected family or caregiver.\n\n A Plan of Safe Care has been shown to reduce child safety risks related to substance use disorders within a family and therefore is an effective strategy for keeping families safely together and preventing the need for foster care. \n\n For infants with prenatal substance exposure, Plans of Safe Care bring together affected families and various service providers — including maternal and infant healthcare, substance use disorder treatment, mental health treatment, early childhood, child welfare. \n\n This collaborative approach ensures a comprehensive response that prevents crisis-based reactions, ensures infant safety, promotes healthy child development, and strengthens family protective factors.\n\n Since 2003, there have been three significant changes in requirements about implementing Plans of Safe Care. There is great variation among states, both in terms of the implementation of their policy and procedures and the rate of infants placed in out-of-home care due to substance use disorder-related issues.\n\n The Keeping Children and Families Safe Act of 2003 created new conditions, including the Plan of Safe Care, for states to receive grant allocations under the Child Abuse and Prevention Treatment Act (CAPTA). The legislation required states receiving a CAPTA grant to implement policies and protocols for the following:\n\n - Appropriate referrals to child protection services and other relevant systems to address the needs of infants born with and identified as affected by illegal substance abuse or withdrawal symptoms resulting from prenatal drug exposure.\n\n - A requirement that health care providers involved in the delivery or care of such infants notify the child protective services agency of the occurrence of such condition except that such notification shall not be construed to establish a definition under federal law of what constitutes child abuse or require prosecution for any illegal action.\n\n - The development of a Plan of Safe Care for the infant born with and identified as being affected by illegal substance abuse or withdrawal symptoms. \n\n You are an expert at searching text for the **EXACT** strings of keywords in {list of keywords from [Appendix Table 1](https://www.sciencedirect.com/science/article/pii/S0145213426002899?via%3Dihub#t0005) piped in here} and returning the **EXACT** sentence and text that the keyword was mentioned in\n\n If a sentence has multiple keywords in it, record the sentence once; do **NOT** record it for every keyword |
| **Prompt** | Please review the following text: {text from APSR page piped in here}\n\n Then return any sentences containing the keywords provided in your system message as an ‘ExtractMentions’ object |
| **ExtractMentions Object** | Pydantic BaseModel object with two attributes:   - 1.   sentences: list of KeywordSentence objects; described to the model as “list of unique KeywordSentence objects which represent sentences containing keywords that you find in this text”   - 2.   summary: string; described to the model as “summary of the text's key points, state actions, or themes related to plans of safe care” |
| **KeywordSentence Object** | Pydantic BaseModel object with three attributes:   - 1.   keyword: keyword chosen from keyword list in [Appendix Table 1](https://www.sciencedirect.com/science/article/pii/S0145213426002899?via%3Dihub#t0005); described to model as “keyword that you found in the sentence; pick the first keyword if there are multiple”   - 2.   sentence: string; described to model as “exact, full sentence containing keyword”   - 3.   context: string; described to model as “exact text, including any relevant surrounding sentences that provide context, containing keyword” |
